# The clinical utility of polygenic risk scores for chronic lymphocytic leukemia

**DOI:** 10.1038/s41375-021-01429-5

**Published:** 2021-09-25

**Authors:** Amit Sud, Philip J. Law, Richard S. Houlston

**Affiliations:** 1 Division of Genetics and Epidemiology, The Institute of Cancer Research, London, UK; 2 Department of Haemato-Oncology, The Royal Marsden Hospital NHS Foundation Trust, London, UK

**Keywords:** Cancer genetics, Cancer epidemiology, Risk factors, Clinical genetics, Haematological cancer

## To the Editor:

We read with interest the article by Kleintsern et al., reporting polygenic risk scores (PRSs) for monoclonal B-cell lymphocytosis (MBL) in Caucasians and for chronic lymphocytic leukaemia (CLL) in African Americans [1]. These data undoubtedly provide further evidence of the polygenic nature of the heritable risk to CLL and its precursor lesion MBL. In their report, which builds on earlier work, they assert that the PRS is a strong predictor of CLL risk, and importantly that the results of their study will aid the identification of individuals at high risk [2]. We believe that assertions regarding the clinical utility of a PRS for CLL need to be tempered. For any PRS to be clinically useful, it firstly needs to provide sufficient risk discrimination and secondly, it must be relevant to strategies for prevention and early detection.

**Fig. 1:**
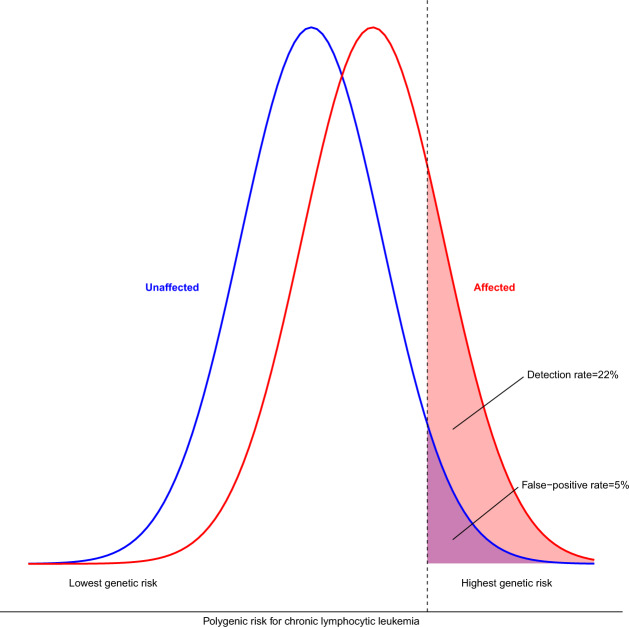
Overlapping relative frequency distributions of polygenic risk scores in chronic lymphocytic leukaemia. The detection rate for a false-positive rate of 5% is 22%.

**Fig. 2:**
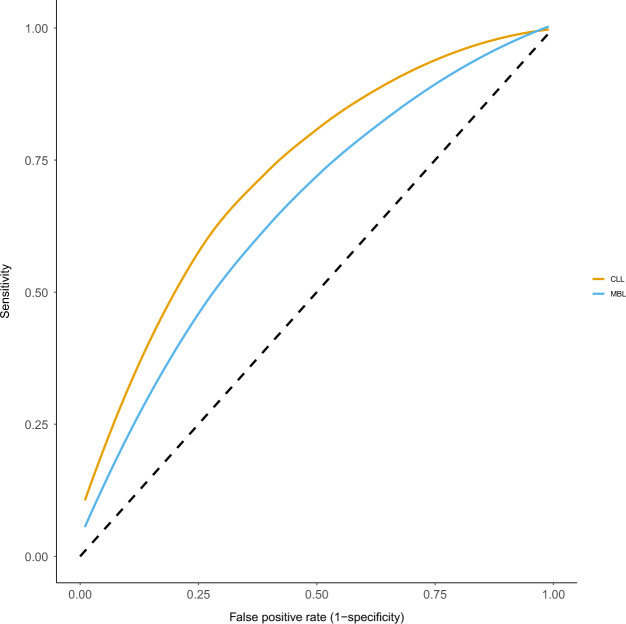
Receiver operator plots of polygenic risk scores for monoclonal B-cell lymphocytosis (MBL) and chronic lymphocytic leukaemia (CLL). Area under the curve (AUC) calculated using the risk-screening converter (https://www.medicalscreeningsociety.com/rsc.asp) and the relative risk estimates between the top and bottom 20% of the population PRS for CLL (orange) and MBL (blue) from Kleinstern et al. [1, 3]. The AUC provides an estimate of the probability that a randomly selected subject with the condition has a test result indicating greater suspicion than that of a randomly chosen individual without the condition. The solid line represents a receiver operator curve based on polygenic risk scores from known risk SNPs. An AUC of 0.5 (dashed line) indicates a classifier that does not differ from the null (i.e. a classifier that does not provide any useful information in discriminating cases from controls).

The risk discrimination provided by PRS is a consequence of the PRS-frequency distributions of those with CLL compared to those without CLL. This can be quantified by: (i) comparison of the relative risk (RR) for those at the top and bottom tails of the PRS distribution (Fig. 1), (ii) comparison for specified PRS cut-offs of the proportion of CLL with ‘positive’ PRS (‘detection rate’ (DR)) versus the proportion of unaffected falling within the same PRS score range (the ‘false-positive rate’ (FPR) (1–specificity)) (Fig. 2) [3]. For CLL, Kleinstern et al. report a RR of 11.7 between the top 20% and bottom 20% of the population PRS, providing an area under the curve (AUC) of 0.72. This translates to a FPR of 5% for a DR of 22%, or, to achieve a DR of 50%, tolerance of a FPR of 19% is required (Fig. 1 and Fig. 2). For MBL, a RR of 5 between the top 20% and bottom 20% of the population is associated with an AUC of 0.65, affording a DR of 50% for a FPR of 28% (Fig. 2). Advocates of PRSs tend to focus its performance on the tails of the distribution, diverting attention from the fact that for 90% of individuals their PRS lies relatively close (<2 standard deviations) to the mean. Modification by these PRSs against baseline risk results in small absolute differences in risk. Furthermore, clinical decision-making is generally driven by absolute risk rather than RR, and for those in the top 20% of PRS, lifetime risk for CLL will be only be elevated from 0.44 to 1.54% (3.5-fold) [1, 4]. Two possible avenues have widely been suggested to improve the predictive capability of PRSs. Firstly, it is argued that the discriminatory value of PRS will improve if additional disease-associated SNPs are identified. Aside from the fact that this is a questionable assertion, the prospects of developing large enough GWAS to harvest 80% of the heritable risk of CLL (requiring a sample size of at least 80,000), seems problematic given that the largest GWAS of CLL so far conducted has been based on only 6200 cases [5, 6]. Secondly, the predictive value of PRS could theoretically be boosted when combined with non-genetic risk factors. However, as the authors point out, there are no known non-genetic factors aside from age, sex and ancestry that have been shown to influence CLL risk. A family history (FH) of CLL is a risk factor but it is correlated with PRS, and models incorporating both FH and PRS result in modest improvements in discrimination [2]. While epidemiology is a dynamic field, it would seem naive to predict imminent discovery of modifiable risk factors for CLL to complement PRSs.

Along with concerns regarding adequate risk discrimination, PRS testing does not currently offer any direct benefit in the prevention or early detection of CLL. Since no modifiable risk factors for CLL exist, the opportunity to reduce an individual’s risk through lifestyle changes is not an option. While chemoprevention of CLL might appear an attractive proposition, no agents are currently licenced for this indication. Moreover, the risk-benefit profile of a therapy informs the level of disease risk at which administration is justified; thus the value of PRS stratification in determining administration is contingent on the performance of PRS in discriminating those at sufficiently elevated risk. The administration of a chemopreventative agent based on PRS-stratified groups has not yet been trialled and would require careful consideration and includes the selection of an appropriate end point such as overall survival (OS). Over the past decades, treatment of early-stage CLL with chlorambucil, fludarabine and combination chemoimmunotherapy has been trialled with no evidence of an OS benefit [7, 8]. With the advent of novel agents such as Bruton’s tyrosine kinase and B-cell lymphoma 2 inhibitors, treatment of early-stage CLL, and particularly of those at high-risk of disease progression, is being revisited [9]. Whilst data from these trials are currently too immature to draw conclusions regarding OS, an increase in adverse events of clinical interest (including atrial fibrillation, bleeding and hypertension) was associated with Ibrutinib therapy when compared to a placebo in the CLL12 trial [9].

It is important to note that in the absence of counselling and appropriate implementation, communicating DNA based disease risk assessments has the potential to incur harm [10]. Currently, when an individual is diagnosed with CLL, one third of patients will never require treatment in their lifetime and an additional third of patients will have an indolent phase followed by disease progression [7]. Given that the PRS implemented by Kleinstern et al. was developed to predict CLL incidence and not morbidity or mortality, the use of PRS in screening for CLL may well raise the issue of over diagnosis.

The GWAS of CLL that have been conducted have undoubtedly informed our understanding of CLL aetiology. However, given the moderate RRs provided by PRS and the lack of efficacious early interventions, the clinical utility of PRS for CLL is currently questionable.
